# Defence of the Doctrine of Vital Affinity against the Objections Stated to It by Humboldt and Dr. Daubeny

**Published:** 1853-01

**Authors:** 


*Defence of the Doctrine of Vital Affinity against the objections stated to it by* HUMBOLDT *and* Dr. DAUBENY. By Dr. ALISON. Communication for the "Examiner" by S. A. LEWIS, M. D., Philadelphia.

The object of this paper was to fix attention on the great physiological discovery which has been gradually effected during the present century, of the mode in which certain of the elements contained in the earth's atmosphere, under the influence of light, and of a certain temperature, are continually employed in maintaining that great vital circulation, of which vegetable structure, animal structures, the air and the soil, are the successive links; and to point out that the most essential and fundamental of the changes here effected—particularly the formation of the different organic compounds in the cells of vegetables—are strictly *chemical changes*, at least as clearly distinct from any chemical actions yet known to take place in inorganic matters, as the vital contractions of muscles are distinct from any merely mechanical causes of motion; and justifying the statement of Dr. Daubeny that there appears to be a "power, residing in living matter," and producing chemical effects, in fact manifesting itself most unequivocally by the chemical changes which result from it, "distinct, at least in its effects, from ordinary chemical and physical forces."

But after having made this statement, Dr. Daubeny, according to the author of this paper, has thrown a degree of mystery over the subject which is quite unnecessary and even unphilosophical, by refusing to admit, and quoting Humboldt, who has changed his opinion upon the subject, and now likewise declines to admit that these changes are to be regarded as *vital*; both authors (as well as several other recent English authors) maintaining that as we do not know all the conditions under which ordinary chemical affinities act in living bodies, we are not entitled to assert that these affinities may not yet be found adequate to the production of the chemical changes which living bodies present; and that until this *negative proposition* is proved, it is unphilosophical and delusive to suppose the existence of any such power as that to which the term vital affinity has been applied by the author of this paper and several other physiologists.

In answer to this, it is here stated, that as we cannot, strictly speaking, *define* life or vitality, we follow the strict rules of philosophy, in *describing* what we call living bodies, whether vegetable or animal, and then applying the term vital or living, as the general expression for everything which is observed to take place only in them, and which is inexplicable by the physical laws, deduced from the observation of the other phenomena of nature ; that according to this, the only definition of which the term vital admits, or by which the objects of Physiology can be defined, Dr. Daubeny has already admitted, in the expressions above quoted from him, that chemical as well as mechanical changes in living bodies fall under the denomination *vital;* and that as the rule of sound logic is "affirmantibus incumbit probatio," and as it is just as probable *à priori*, that with a view to the great objects of the introduction of living beings upon the earth, the laws of chemistry, as those of mechanics, should be modified or suspended by Almighty Power, this author maintains that we are as fully justified in referring all great essential chemical phenomena which are peculiar to living bodies, to peculiar affinities which we term vital, as Haller was to ascribe the peculiar mechanical movements of living bodies to the vital property of irritation ; and to throw on the mechanical physiologists of his day the burden of proving, if they could, that the laws of motion perceived in dead matter were adequate to explain them.

In illustration of the importance, both in Physiology and Pathology, of this principle being held to be established, Dr. Alison adduced two examples, *first*, the utter failure of the very ingenious theory of Dr. Murray to explain, on ordinary chemical principles, the simplest and most essential phenomena of healthy secretion ; and, *secondly*, the now generally admitted inadequacy of any theory of inflammation, which does not regard a modification of the *affinities* peculiar to life, and here termed vital, as the primary and essential change, in the matter concerned in that process.*

Dr. Alison's paper may be seen *in extenso*, in the Edinburgh Ph. Trans. 1851–52.

* Jamieson, Edinburgh N. P. Journal, October, 1852.

